# Supplementary material for: Cylindrical Microparticles Composed of Mesoporous Silica Nanoparticles for the Targeted Delivery of a Small Molecule and a Macromolecular Drug to the Lungs: Exemplified with Curcumin and siRNA
Source: Pharmaceutics. 2021 Jun 7;13(6):844. doi: 10.3390/pharmaceutics13060844 (PMC8230201; doi:10.3390/pharmaceutics13060844)
Supplement: Supplementary file 1 [file pharmaceutics-13-00844-s001.zip › pharmaceutics-1241581-supplementary.pdf]

pharmaceutics

Supplementary Materials: Cylindrical Microparticles Composed of Mesoporous Silica Nanoparticles for the Targeted Delivery of a Small Molecule and a Macromolecular Drug to the Lungs: Exemplified with Curcumin and siRNA

Thorben Fischer, Inga Winter, Robert Drumm and Marc Schneider

1. Transmission Electron Microscopy

Transmission electron microscopy (TEM) is a special form of electron microscopy that enables high magnification of small objects (nanometer scale) [1]. Here, electron beams are used to produce a direct image of the sample. Electrons emitted from the cathode are accelerated up to 200 keV. In contrast to scanning electron microscopy, here one detects the electrons passing through the samples. A condenser lens system focuses the electron beam onto the specimen plane. Subsequently, an object lens produces a slightly magnified intermediate image, which is further magnified by subsequent lenses (diffractive, intermediate and objective lenses) [2].

For sample preparation, the sample was applied to the sample carrier (TEM grid) and contrasted. For this purpose, the TEM grid was first immersed for 45 s with the sample side down in an aqueous 2% uranyl acetate solution. After 2 min exposure time, the sample was cleaned with ultrapure water in a washing step. After the sample was completely dry, it was clamped in the sample holder and examined by transmission electron microscopy at a magnification of 20 kX and 50 kX.

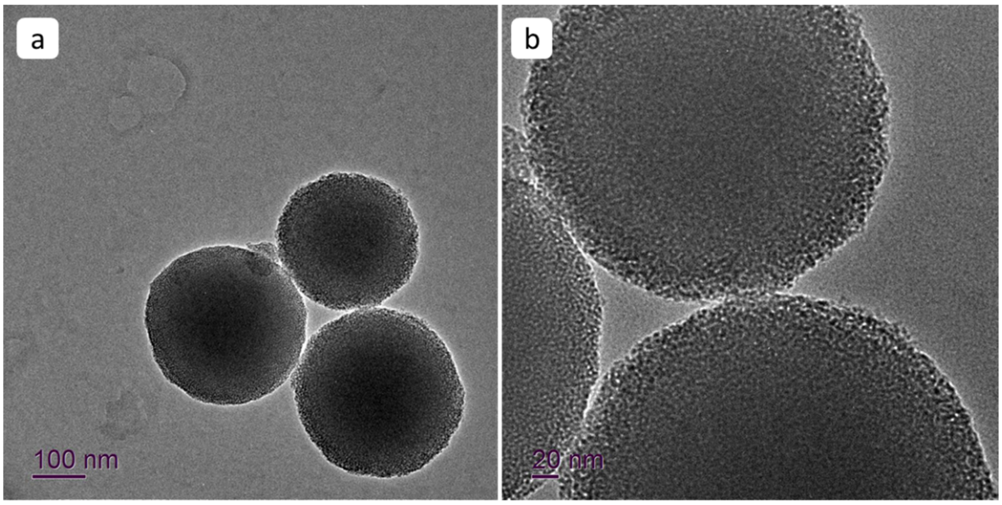

Figure S1. TEM images of the mSNP, visualizing the mesoporous structure. Both magnifications (**a**) 20 kX and (**b**) 50 kX illustrate the sponge-like strucutre, which indicates a large pore volume to load high amounts of drug.

2. Experimental Determination of the Loading Amount of Mesoporous Silica Nanoparticles

To determine the maximum loading capacity of the mSNPs with curcumin, the nanoparti-cles were loaded with different amounts between 5 to 20% and the total pore volume was determined by BET measurement (Figure S2). The results obtained show that by increasing the loading amount, the total pore volume is decreasing. At the highest loading tested (20%), there is only a small free pore volume of 0.0298 cm3/g left, which

correlates with the calculated maximum loading of approx. 22%. For this reason, a curcumin loading of 20% was selected in the experiments performed in the manuscript.

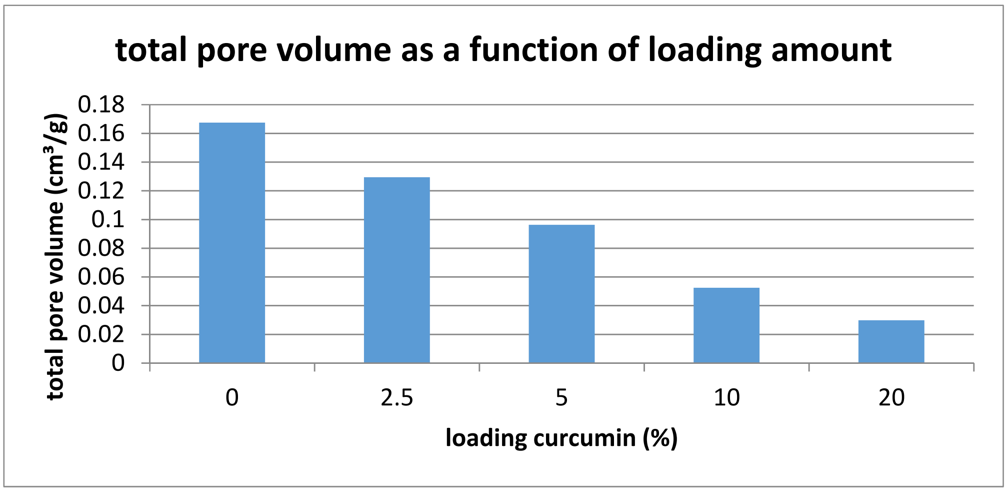

Figure S2. Correlation between total pore volume and loading amount of curcumin. The experimental determination of the maximum loading amount shows that at a loading of 20% almost all pores are filled. This also corresponds to the calculated maximum pore loading of approx. 22%. For each loading amount, two individual measurements were performed ($n = 2$).

3. Functionalization of the mSNPs with Rhodamine B and Quantification

After the particles were functionalized with rhodamine B, a calibration curve was created using microplate spectrophotometer to both establish a correlation between emission and concentration and confirm successful functionalization.

The calibration curve plotted in Figure S3 shows that staining with rhodamine B was successful. Furthermore, the linear relationship between concentration and the fluorescence signal (at $\lambda_{ex} = 545$ nm, $\lambda_{em} = 570$ nm) allows quantification.

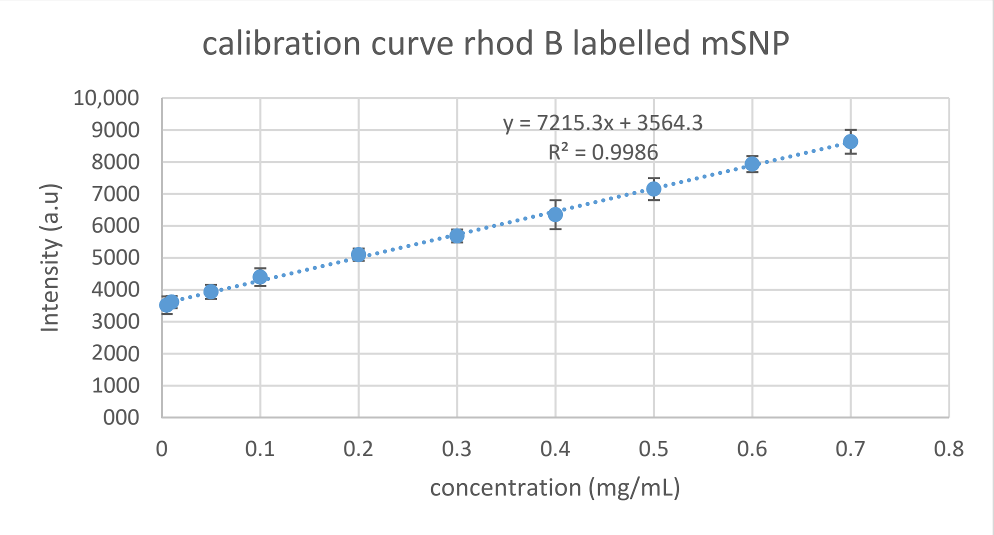

Figure S3. Calibration curve of mSNPS functionalized with rhodamine B at $\lambda_{ex} = 545$ nm, $\lambda_{em} = 570$ nm. For each measuring point, three individual measurements were performed ($n = 3$).

References

1. Inkson, B. Scanning electron microscopy (SEM) and transmission electron microscopy (TEM) for materials characterization. In *Materials Characterization Using Nondestructive Evaluation (NDE) Methods*; Elsevier: Amsterdam, The Netherlands,2016; pp. 17–43.
2. Reimer, L. *Transmission Electron Microscopy: Physics of Image Formation and Microanalysis*; Springer: Berlin/Heidelberg, Germany, 2013; Volume 36.
